# Visual Improvement Following Stereotactic Radiosurgery for Orbital Apex Vascular Tumor

**DOI:** 10.7759/cureus.75702

**Published:** 2024-12-14

**Authors:** Georgios Chondrozoumakis, Kosmas Verigos, Efstathios Detorakis

**Affiliations:** 1 Department of Ophthalmology, University General Hospital of Heraklion, Heraklion, GRC; 2 Department of Radiotherapy, 401 General Military Hospital of Athens, Athens, GRC

**Keywords:** cavernous hemangioma, orbital apex, orbital tumor, orbital vascular lesion, stereotactic radiosurgery

## Abstract

Orbital apex lesions represent a clinical challenge since they are difficult to remove surgically and may induce significant functional defects. The orbital apex is an area of convergence of neurovascular elements passing through the various local osseous foramina and the congestion of several critical anatomical structures in a confined space increases the risk of intraoperative complications. Radiotherapy is an alternative treatment option in such cases but may also induce radiation toxicity. We present a case of a vascular orbital apex lesion, which caused vision loss and was treated with stereotactic radiosurgery, resulting in significant visual restoration.

## Introduction

Surgical access to the orbital apex can be achieved through various pathways in surgical anatomy, including a lateral orbitotomy, medial transconjunctival orbitotomy, or transpalpebral or lid-split anterior orbitotomy [1]. In all cases, however, there is an increased risk of iatrogenic damage to critical structures, such as the optic nerve, autonomous innervation of the eye, oculomotor apparatus, or the superior orbital vein [2]. The decision to perform surgery in the orbital apex relies on the presence of lesions that may be malignant (thus requiring incisional or excisional biopsy) or benign but producing functional defects through chronic pressure on anatomical structures, such as the optic nerve [3]. In fact, compartment syndrome may arise in the orbital apex resulting in compressive optic neuropathy even by benign lesions, such as cavernous hemangiomas, located at the orbital apex [4].

Since surgery may result in further loss of residual vision, alternative treatment options, such as radiotherapy, should be considered [5]. Conventional radiotherapy may be effective in treating orbital vascular lesions but the therapeutic result is compromised by collateral damage to surrounding anatomical structures [6]. Stereotactic radiosurgery (SRS) may overcome this limitation by concentrating the treatment dosage on a small target, such as the tumor, sparing adjacent structures [7]. Previous studies have reported successful outcomes with a four-dose fractionated SRS treatment.

We present a case of an orbital apex vascular lesion successfully treated with a three-dose fractionated SRS, which resulted in a significant reversal of vision loss. The main purpose of this case report is to highlight the effectiveness of radiotherapy in challenging cases when the function of the optic nerve is imperiled.

## Case presentation

A 57-year-old man was referred to our practice for a reported progressive painless reduction in vision in his left eye. Systemic history was non-contributory. The patient was a non-smoker and did not report regular use of medications or drugs. Previous ophthalmic history was also non-contributory. Upon presentation, the best corrected visual acuity (BCVA) of his right eye was 10/10 and of his left eye 6/10. A Snellen optotype was used for all the measurements of visual acuity. Intraocular pressure was 12 mmHg in both eyes (OU) (without medications). Anterior segment biomicroscopy and fundoscopy were non-contributory. Pupillary motility examination was significant for left relative afferent pupillary defect (RAPD). Color vision examination revealed impaired color perception (Ishihara plates score = 7/24).

**Figure 1:**
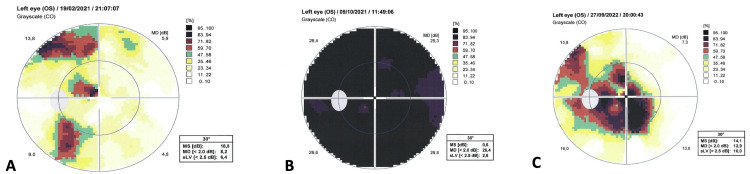
Series of 30-degree visual fields of the left eye, including the initial presentation (A), deterioration four months later (B), and improvement following stereotactic radiosurgery (C).

**Figure 2:**
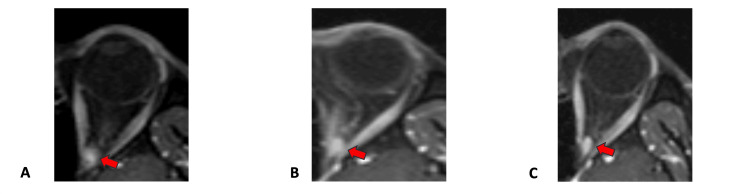
Series of transverse T-1 oriented, fat-suppressed, and contrast-enhanced orbital MRI scans. The orbital apex lesion is shown with red arrows at first presentation (A), four-month follow-up interval (B), and 12 months post stereotactic radiosurgery (C), demonstrating a slight reduction in size.

Automated visual field examination (Octopus 900, Haag-Streit Diagnostics, Köniz, Switzerland) revealed a -8.2 dB mean defect (MD) to the sensitivity to light in his left eye (the respective score of the right eye was -1.8 dB). The distribution of reduced sensitivity to light in the visual field of the left eye (OS) was non-specific (Figure 1A). An MRI scan of the orbits with thin sections (1 mm) was then prescribed, which was significant for a small mass located at the apex of the left orbit, in close proximity to the optic foramen (Figure 2A). The lesion was clearly delineated and a significant contrast enhancement was observed, implying that the mass was highly vascular.

A working diagnosis of an orbital apex hemangioma was then suggested and surgery was offered. The risks associated with surgical removal were presented to the patient who opted for close observation with the possibility of treatment upon deterioration of vision. Indeed, four months later, the patient reported further progression in vision loss in his left eye (with a recorded visual acuity of 1/20 cc and an MD of -28.4 dB, Figure 1B).

Another MRI scan of the area revealed a modest increase in the size of the offending lesion. Tumor volume, measured by planimetry (manual delineation of tumor borders to calculate the area occupied by the tumor in each section of visible tumor and multiplying by the number of respective sections and section thickness) was 268.4 mm^3^ and 306.2 mm^3^ at first presentation and four-month follow-up interval, respectively (Figure 2B).

**Figure 3:**
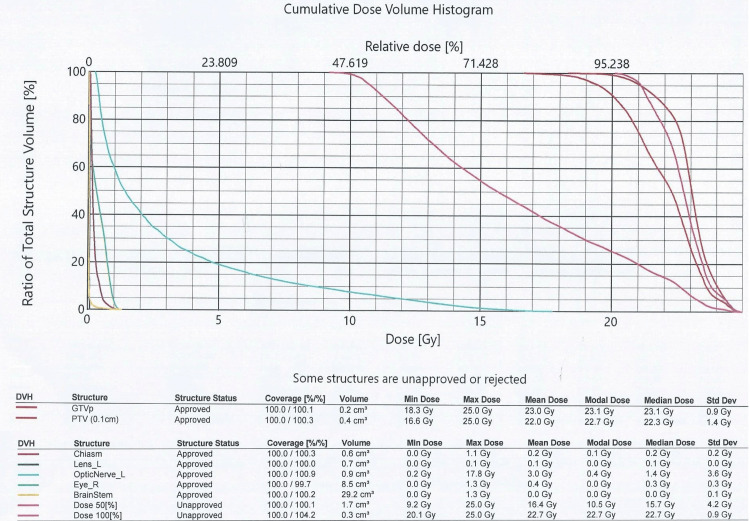
Cumulative dose-volume histogram. Dose delivered to adjacent structures, such as the optic chiasm, lens, and optic nerve. DVH: dose-volume histogram; GTV: gross tumor volume; PTV: planning target volume. This image is the original work of the authors.

Treatment options were discussed, taking into account the potential treatment-related side effects on ocular and pupil motility despite the fact that the chances of visual restoration were considered minimal. Accordingly, the option of SRS was offered to minimize potential radiation toxicity to the adjacent tissues. The patient underwent SRS of the lesion at the Department of Radiotherapy of 401 General Army Hospital in Athens Greece with TrueBeam linear accelerator (Varian, Palo Alto, CA). The total dose delivered to the lesion was 21 Gy, fractionated in three sessions of 7 Gy, based on previous reports on lesions of similar size and location [8]. Planning dosimetry with dose-volume histogram is presented in Figure 3. After radiotherapy, the patient was closely followed to monitor the size of the lesion as well as any radiation-associated effects. BCVA of the left eye gradually increased to 2/10 (six months post irradiation) and 4/10 (12 months post irradiation), with associated significant improvement (compared to the immediate pre-radiation levels) in the MD score of the visual fields to -12.9 dB (Figure 1C). Moreover, the resolution of the RAPD and improvement of color perception (post-SRS Ishihara score of 14/24) were noted. A post-SRS MRI scan of the orbits confirmed a measurable decrease in the tumor size at 273.3 mm^3^ (Figure 2C). No other clinically significant side effects from the application of SRS, including ocular motility or pupillomotor effects, were observed.

## Discussion

Orbital apex syndrome is characterized by optic nerve dysfunction, which may be accompanied by ocular motility deficits as well as periocular pain due to involvement of the V1 trigeminal branch [4]. Superior orbital fissure and cavernous sinus syndromes exhibit overlying clinical features with orbital apex syndrome, hence they constitute the main differential diagnosis. However, the first typically spares optic nerve function, whereas the latter concurrently affects the maxillary division of the trigeminal nerve [4].

Orbital cavernous hemangioma (OCH) is the most common benign orbital neoplasm in adults [9]. Despite intraconal space being the most common primary location, cavernous hemangiomas can arise anywhere within the orbit. Orbital apex can also be the presentation site, potentially giving rise to symptoms even with lesions of lesser volume, due to the tight proximity of important anatomical structures [9].­ Diagnosis can be set accurately on the basis of MRI findings, differentiating cavernous hemangiomas from miscellaneous neoplastic and vascular tumors, by highlighting differences in signal intensity and contrast enhancement [10].

Numerous studies have demonstrated the role of surgical resection of OCHs in imperiling optic nerve function [11-13]. The surgical approach used to be the mainstay of treatment, especially for lesions easily anatomically accessible. However, lesions adjacent to the orbital apex are correlated with a higher rate of complications [12,14]. Multidisciplinary surgical management is often required in such cases, utilizing, apart from standard orbitotomy, transcranial and transnasal approaches [13]. The present report implies that SRS provides a valid therapeutic alternative, with equivalent functional outcomes, while the iatrogenic damage can be minimized with meticulous preoperative planning.

In cases of recent origin, the reduction in tumor size following SRS may enable partial or complete restoration of the optic nerve function, when optic disc pallor, implying optic nerve atrophy, has not been established yet [15]. Not only is this achieved by direct reduction in tumor size, but also due to other unknown pathophysiologic factors. Indeed, there have been several reports of dramatic improvement in visual acuity not accompanied by the expected reduction of the tumor size [15]. Orbital apex vascular tumors can compromise vision by either direct infiltration of the ipsilateral optic nerve or by compressive action on the nerve stem (compressive optic neuropathy) [15]. However, in long-standing cases, optic atrophy ensues, implying that vision loss may be irreversible [8].

SRS implements external beams of radiation precisely focused on the target, composed of either photons or charged particles, such as protons. Gamma Knife and high-energy X-rays are the two most widely available, non-particle options. The total dosage can be delivered in one session or fractionated over multiple ones [7,15]. Multi-sessions of fractionated Gamma Knife-based radiotherapy are more difficult in execution, as in most cases a framework is fixated to the patient’s head, which is required to remain placed for the whole treatment period. However, frameless Gamma Knife has also been an option since 2016 for SRS of CNS tumors [8].

Hereby, we utilized high-energy X-rays delivered by a linear accelerator (LINAC) aimed at containing the local effect of an OCH of the orbital apex. A fractionated SRS protocol was followed to avoid excessive single dosage irradiation of the optic nerve, while still achieving the overall cumulative dosage required for the desired cytotoxic effect on the tumor cells. While previous studies have employed a four-fraction protocol, in this case, a three-fraction protocol was used, implying that the therapeutic target in such cases can be achieved with a less fragmented protocol without compromising treatment efficacy and safety [8]. It has been proposed that radiation-induced neuropathy is less frequent at dosages less than 10 Gy and cavernous hemangiomas response to the therapy ranges between dosages of 16 and 20 Gy [15].

A previous study by Sasaki et al. of five patients with affected visual function due to OCHs has demonstrated a tumor response following CyberKnife (Accuray Inc., Sunnyvale, CA), which is also a high-energy X-ray-based SRS, implementing marginal dosages of approximately 25 Gy [16]. All patients exhibited visual improvement, while one SRS resulted in further visual deterioration. Nevertheless, detailed visual function progression was not disclosed in this study [16].

Α 2018 study by Young et al. has also reported success in the management of OCHs by applying four doses of Gamma Knife-based SRS, 5 Gy each, thus achieving a cumulative dose of 20 Gy, similar to our treatment planning, but, as previously mentioned, with four, instead three, fractions [8]. Ten out of 12 patients with compressive neuropathy exhibited visual function improvement along with tumor shrinkage, whereas the rest remained clinically unchanged due to long-standing atrophy. Our results are in accordance with the findings of Young et al., indicating that OCHs display highly radiosensitive features. These characteristics imply that patients with OCHs are appropriate candidates for SRS. Other orbital tumors such as meningiomas of the optic nerve sheath are less sensitive, requiring higher total dosages of 50 Gy, thus making the decision for radiotherapy more complex [17].

Two previous reports from Liu et al. and Khan et al. demonstrated results after a single session of Gamma Knife SRS of 23 patients and one patient with OCH and visual impairment, respectively [18,19]. Anatomical tumor regression was reported, even though the total tumor dosage was maintained below 15 Gy to achieve the minimum possible side exposure of the optic nerve. However, no detailed follow-up results regarding the visual function are available from both studies.

## Conclusions

In conclusion, this case report highlights the efficacy of SRS against a vascular orbital apex lesion (presumed OCH), which had already affected optic nerve function even with a reduced number of treatment fractions. Moreover, it provides evidence for partial recovery of optic nerve function following the successful completion of SRS. The lack of histopathological documentation for a definitive diagnosis stands as a main limitation in our study, albeit it is an inevitable disadvantage of non-invasive approaches. On the contrary, our main objective is to present SRS of such lesions as an equally efficient solution to the surgery, yet with potentially fewer complications.

On the other hand, we provide data about LINAC-delivered SRS, comparable to the Gamma Knife studies, both in terms of optic function restoration, as well as in overall safety. Fractionation of the therapy also becomes a more feasible option with LINAC. To our knowledge, this is the first report providing detailed data about the visual recovery following high-energy X-ray SRS of an orbital apex OCH. However, future comparative studies may be required to determine standardized treatment protocols, depending on the available modality, for optimal and safe results in the therapy of benign orbital tumors of vascular origin.
